# Age at Menarche and Menstrual Cycle Pattern among School Adolescent Girls in Central India

**DOI:** 10.5539/gjhs.v4n1p105

**Published:** 2012-01-01

**Authors:** Dharampal G. Dambhare, Sanjay V. Wagh, Jayesh Y. Dudhe

**Affiliations:** Department of Community Medicine, Mahatma Gandhi Institute of Medical Sciences Wardha, Maharashtra, India Tel: 91-098-8157-7698 E-mail: darampal21@yahoo.com; Department of Preventive and Social Medicine Government Medical College Akola – 444001, Maharashtra, India Tel: 91-090-9654-0650 E-mail:drsanjayvwagh@rediffmail.com; Department of Preventive and Social Medicine Government Medical College Akola – 444001, Maharashtra, India Tel: 91-097-6705-9587 E-mail: jayeshdudhe@gmail.com

**Keywords:** Age at menarche, Menstrual abnormalities, Socio-economic class

## Abstract

**Background:**

The onset of menstruation is part of the maturation process. However, variability in menstrual cycle characteristics and menstrual disorders are common. The purpose of this study was to determine the age at menarche and patterns of menstruation among school adolescent girls and explore its variation across socio-economic and demographic factors.

**Methodology:**

This is a cross-sectional descriptive study carried out on 1100 school adolescent girls in district Wardha, Central India. Data were collected using a self-administered structured questionnaire on menstruation. Data was entered and analyzed by using Epi Info 6.04 software package. Chi- square value was used for testing statistical significance.

**Results:**

Mean ages of menarche were 13.51 + 1.04 years and 13.67 + 0.8 years for urban and rural areas respectively. Abnormal cycle length was common and affected 30.48%. The majority 56.15 experienced dysmenorrhoea and 56.16 percent had premenstrual syndrome. Self medication was practiced by 7.13% of the adolescent girls. The most common premenstrual symptom was headache 26.74%. Absenteeism from the school 13.9% was the effect of menstruation related problems on their daily routine. Dysmenorrhea and premenstrual symptoms were perceived as most distressing symptoms leading to school absenteeism. Majority of the girls 75.58% had discussed menstrual problems with someone, most commonly with their mothers 38.15%. There was a general lack of information about menstrual issues especially with regards to cycle length, duration of menses and age at menarche. Girls from families of high socio-economic class have significantly lower mean menarcheal age in both urban and rural area. The mean age of menarche was significantly higher in girls involved in vigorous sporting activity in urban area compared to their non-sporting counterparts.

**Conclusion:**

Age at menarche was delayed. The menstrual disorders among female adolescents are common. A school health education on menstrual problems targeting adolescent girls and their parents and routine screening for menstrual problems by healthcare providers can help to prevent the absenteeism in the school.

## 1. Introduction

Adolescence in girls has been recognized as a special period which signifies the transition from girlhood to womanhood. This transitional period is marked with the onset of menarche, an important milestone. Menstruation is a normal physiological process that begins during adolescence and may be associated with various symptoms occurring before or during the menstrual flow. Adolescent girls constitute a vulnerable group, particularly in India where female child is neglected one. Menarche is a part of the complex process of growing up. The age of onset of menstruation varies from 9 to 18 years with the average age in United States being about 12 years and 8 months, whereas in India it is slightly lower and has been reported to be around 12 years (Khadilkar VV *et al* 2006, Chumlea WC *et al* 2003). The age at menarche shows many socioeconomic, environmental, nutritional and geographical differences in the societies (Attallah NL 1978, Ekele BA *et al* 1996).

These problems include psychological adjustment with menstruation, premenstrual and menstrual symptoms and disorders of menstruation. Female experience premenstrual symptoms 7 to 10 days before the onset of bleeding. These include irritability, malaise, headache, acne, abdominal pain etc. the main importance of the premenstrual tension is psychosomatic. The menstruation in majority of female is asymptomatic apart from per vaginal bleeding, however some may have pain in abdomen with or without gastroinstestinal upsets like anorexia and vomiting (Padubidri VN *et al* 1997). Complaints like leg pain, backache may also be associated with normal menstrual cycle (Banerjee D *et al* 1961). The medical and social consequences of premenstrual, menstrual symptoms and disorders of menstruation influence not only the individual but also her family and society. In respect to adolescent girls it may manifest as loss of school days leading to poor progress in education. This may lead to problems in continuation of her education (Deo D S *et al* 2007a). However few studies in India have described the lifestyle factors associated with various menstrual cycle patterns. The present study, therefore, aims to determine the age at menarche and patterns of menstruation among school adolescent girls and explore its variation across socio-economic and demographic factors.

## 2. Material and Methods

A cross sectional study was conducted among school adolescent girls of classes five to twelve in the District Wardha, Maharashtra, Central India between October to December 2009. The girls were selected according to WHO criteria for the adolescence that is 10 – 19 years (World Health Organization 1984). Random selection of 6 schools and 1100 girls from these schools were enrolled, three rural and three urban schools were surveyed. Using the retrospective method, about 1100 questionnaires were distributed, 1080 were filled correctly. Of this, 519 (48.1%) girls did not yet experience their first menstruation at the time of interview and these students were not included in the study, so we had 390 and 171 answered questionnaires from urban and rural schools respectively. Effort was made to examine the students who were absent on a particular day at the next visit. All the girls were interviewed by the team comprising of doctor, nurse, social worker and school teacher through a scheduled visits. Informed consent of the school adolescent girls and head of the institution was taken for conducting the study. Due approval was obtained from institutional ethical committee.

A pre-designed, pre-tested questionnaire was used for data collection. The questions was administered in local language (*Marathi*) and properly explained to avoid any form of misunderstanding and to facilitate accurate response by the subjects. The questionnaires distributed and collected immediately after completion to prevent interpersonal communication and influence of peers on individual responses amongst the girls. The information was gathered on sociodemographic data, menarcheal age, menstrual cycle pattern, premenstrual symptoms, dysmenorrhea and associated symptoms. The age at menarche was determined by questioning the school girl if she had or not had menstruation and at which age she had menstruated. Data thus generated was entered and analyzed using Epi Info 6.04 software package. Means, standard deviations and simple percentage were determined. Chi- square value was used for significance level. P < 0.05 was considered significant.

## 3. Results

Of the 1100 questionnaires distributed 1080 were filled correctly (98.18%). 324 adolescent girls were of rural residence while 756 were urban ones. 519 (48.1%) girls did not yet experience their first menstruation at the time of interview and these students were not included in the analysis of age at menarche, menstrual pattern and disorders.

The age of the school girls interviewed in this study was ranges between 10 to 19 with mean and standard deviation of 15.45 ± 1.75 years. The mean age and standard deviation at menarche was 13.67± 0.8 years. Out of the total study population, 47 (8.38%) adolescents had a menstrual cycle length shorter than 21 days, 390 (69.52%) had a cycle length between 21 and 35 days and 124 (22.1%) longer than 35 days. There was no statistically significant difference on cycle length among the subjects living in urban or rural residence. The mean duration of flow was 4 ± 0.8 days (Table 1).

**Table 1:** Menstrual characteristics of school girls

Menstrual characteristics	No	Percentage
Duration of flow (days)		
< 2	9	1.6
2 - 4	379	67.56
5 - 7	166	29.59
> 8	7	1.25
Cycle length		
< 20 days	47	8.38
21 - 35 days	390	69.52
36 – 45 days	77	13.73
> 45 days	47	8.38

The subjects living in urban areas revealed a mean and median age at menarche of 13.51 and 14.0 years respectively. The subjects living in rural areas revealed a mean and median age at menarche of 13.67 and 14.0 years respectively. The mean age at menarche was 0.16 years younger for urban girls compared to rural area which is not statistically significant. The menstrual cycles were regular in 70 % of the urban and 68.42% rural subjects. There was no statistically significant difference of menstrual cycle regularity by urban or rural residence and age of menarche.

The overall prevalence of dysmenorrhoea was 56.15%. Dysmenorrhoea was significantly more frequent among students from urban as compared with rural residence (Table 2). However, dysmenorrhoea was not significantly associated with age at menarche. Dysmenorrhoea resulted in absence from school of 78 (24.76%) girls with dysmenorrhoea. There was a significant association between the severity of dysmenorrhoea and the duration of menstrual flow.

**Table 2:** Menstrual cycle pattern of the school girls in urban and rural area

Variables	Urban (N-390)	Percentages	Rural (N-171)	Percentages	P value
Age at menarche	13.51 + 1.04		13.67 + 0.8		>0.05
Regular cycles	273	70.00	117	68.42	>0.05
Dysmenorrhea	237	60.77	78	45.61	<0.05
Premenstrual symptom	203	52.05	113	65.50	<0.05
Use of self medicine	29	7.44	11	6.43	>0.05
School absence	47	12.05	31	18.13	<0.05

Dysmenorrhea was more frequently observed among adolescents with irregular cycle (57.9%) as compared to those with regular cycle (42.1%) p < 0.05. 7.13% of the adolescents girls use analgesics to relieve the pain. Premenstrual symptoms were present in 56.15% girls. The most common symptom was headache which was experienced by 26.74%. There was statistically significant association between presence of premenstrual symptoms and rural residence. Self medication was practiced by 7.13% of the adolescent girls (Table 2).

School girls from high socio-economic class had significantly lower mean menarcheal age compared to girls from low socio-economic class in both urban and rural area (p value <0.05, Table 3).

**Table 3:** Age at menarche and socio-economic status of school adolescent girls

Residence	High Mean+SD	Middle Mean+SD	Low Mean+SD	High Vs Low p value	Middle Vs Low p value
Urban area	12.89 + 1.22 (N-27)	13.62 + 0.95 (N-270)	13.48 + 1.35 (N-93)	<0.05	>0.05
Rural area	12.90 + 1.27 (N-27)	13.71 + 0.69 (N-114)	13.63 + 0.68 (N-30)	<0.05	>0.05

(Socio-economic status: High = Rs. 10000 and more family income per month; Middle = Rs. 3000 to 9999 family income per month; Low = less than Rs. 3000 family income per month)

In urban area, school girls involved in vigorous sport activity had a significantly higher age of menarche as compared to girls in non sport activity (p value <0.05, Table 4).

**Table 4:** Relationship between menarcheal age and sport activity

Residence	Sport Mean+SD	Non-Sport Mean+SD	p value
Urban (N-390)	13.77 + 0.95 (N-51)	13.4 + 1.11 (N-339)	<0.05
Rural (N-171)	13.72 + 0.7 (N-108)	13.57 + 0.96 (N-63)	>0.05

(sport activities five hours per week: swimming, volleyball, tennis)

24.42% of the adolescents had no information prior to the commencement of menstruation. 38.15% study subjects had got information about menarche from the mother, followed by friends (32.26%), teachers (3.03%) and books or magazines 2.14% (Table 5).

**Table 5:** Source of premenarcheal information

Source	Total	Percentages
Mother	214	38.15
Friends	181	32.26
Teachers	17	3.03
Book/Magazines	12	2.14
None	137	24.42

## 4. Discussion

Menstruation though a normal physiological process is many a time associated with premenstrual and menstrual disturbances. These disturbances may sometimes be very severe leading to loss of work days.

In the present study, the mean age of menarche of the adolescent school girls was 13.67 years whereas various studies conducted in Kalamboli the mean age at menarche was found to be 13.32 years, in West Bengal 12.8 years and in Turkey 12.81 years (Nemade D *et al*. 2009, Dasgupta A *et al*. 2008 & Demir SC *et al*. 2000).

The subjects living in urban area revealed 13.51 mean age at menarche as compared with 13.67 from rural area. This is consistent with the studies conducted in China and Nigeria (Lin WS *et al*. 1992, Ikaraoha CI *et al*. 2005).

In the present study, the intermenstrual interval was reported to be 21-35 days by 69.52% girls whereas it was 36-45 days for 13.73% girls and more than 45 days for 8.38% girls. This could be because of changing trends in lifestyle, dietary habit, stress, hormonal imbalance or some medical reasons which requires gynaecological assessment at the earliest. In a study conducted among tribal Gujjar adolescent girls, 9.9 per cent of the subjects had their menstrual cycle between 45-60 days which is similar to the figure in the present study (Dhingra R *et al* 2009).

30.48% adolescent girls reported that their cycles were irregular. Irregular cycles are common in adolescents as the initial cycles are anovulatory resulting in abnormal uterine bleeding that may be associated with varying amount of blood loss including menorrhagia (Lee HK *et al*. 2006).

The mean duration of menstrual flow was 4 days, whereas in the study conducted in Turkey the menstrual flow lasting more than 8 days (Sule ST *et al*. 2007).

56.15% of the girls had dysmenorrhoea, whereas majority 61.27% of the girls reported dysmenorrhoea in the study conducted in Dharan and Turkey and in urban area 65% (Houston AM *et al*. 2006, Sharma M *et al*. 2003).

Dysmenorrhoea was significantly associated with higher age most likely because menstrual cycles are commonly anovulatory for some time after puberty whereas dysmenorrhoea is associated with ovulatory cycles (Odujinrin OM *et al* 1991). A similar association between dysmenorrhoea and older age has been reported previously (El-Gilany AH *et al*. 2005, Jacks TH *et al*. 2005).

56.16% of the girls had premenstrual symptoms. Headache, fatigue, feeling of increased weight, abdominal bloating, backache, breast heaviness and joint pain were the most common pre-menstrual symptoms experienced by the adolescent girls. Premenstrual symptom has been reported to be one of the most distressing problems associated with menstrual cycle. The complaints of the adolescent girls in the present study were well within the range as reported by other studies (Banikarim C *et al.*, 2000, Joseph GA *et al.*, 1997).

Absenteeism 13.9% from the school was the effect of menstruation related problems on their daily routine. Dysmenorrhea and premenstrual symptoms were perceived as most distressing symptoms leading to school absenteeism. Dysmenorrhea and premenstrual symptoms has also been reported to be one of the most frequent causes of absenteeism from school and of days off work (Drife JO *et al.*, 2004). Women with premenstrual symptoms have reported a greater number of days with impairment in routine work, school and household activities (Dean BB *et al.*, 2004, Robinson RL *et al.*, 2000).

The study results show the disturbance of routine activities of the study subjects due to dysmenorrhea and premenstrual symptoms (Banikarim C *et al.*, 2000, Busch CM *et al.*, 1988). The rate of school absenteeism is slightly lower than reported by other studies this can be explained by responses to various gradients of pain.

Self medication was practiced by 7.13% of the adolescent girls. However, the study conducted at Turkey reported that 56.60% of the adolescent girls practiced self medication (Sule S T *et al.*, 2007).

School girls from a high socio-economic class had a statistically significant lower mean age of menarche compared to those in low socioeconomic class in both urban and rural area. This is consistent with the finding reported by other studies (Ikaraoha CI *et al.*, 2005, Ikechebelu JI, 1991).

In urban area, school girls involved in vigorous sport activity had a significantly higher age of menarche as compared to girls in non sport activity. Similar findings were reported from other studies (Ikaraoha CI *et al.*, 2005, Stager JM *et al.*, 1988).

24.42% of the adolescents had no information prior to the commencement of menstruation. There was general lack of accurate information about all menstrual abnormalities among the girls in urban as well as rural girls. Previous researchers have also found that knowledge about menstrual abnormalities was very poor among adolescent school girls (Sharma M *et al.*, 2003).

Though there was no significant difference regarding awareness in urban and rural girls their source of information varied. In the urban study group mother was the main source of information. This might be due to better literacy level of the mother and better relation among mother and daughter. Friends were the most common source of information in rural girls. However, in urban girls the source of information about menstruation was their mother 27.5% while it was teacher 27.01% in the rural counterparts (Deo DS *et al.*, 2005b). It was also observed that adolescent girls lacked conceptual clarity about menstruation. The reason was that they had no prior information about menstruation due to which they faced several problems about menstruation and related reproductive health issues.

## 5. Conclusion

In conclusion, the age at menarche was delayed. The menstrual disorders among female adolescents were common. A school health education on menstrual problems targeting female adolescents and their parents, and routine screening for menstrual problems by healthcare providers, can help to prevent the absenteeism in the school.
